# Multi-scenario datasets for system identification and adaptive control of a nonlinear two tank system

**DOI:** 10.1038/s41597-026-07686-2

**Published:** 2026-06-30

**Authors:** Pedro-Pablo Gómez-González, Álvaro Michelena, Agustín García Fischer, Noel Freire-Mahía, Esteban Jove, José Luis Calvo-Rolle

**Affiliations:** Research Group CTC, CITIC, Department of Industrial Engineering, University of A Coruña, Ferrol, 15403 A Coruña Spain

## Abstract

Experimental datasets are essential for validating adaptive control strategies, data-driven monitoring approaches, and control-oriented system identification methods. Despite their importance, publicly available benchmarks based on real nonlinear processes remain limited. This work presents a collection of 25 long-duration time-series datasets totaling over 760,000 samples, obtained from a laboratory two tank water pumping plant. Experiments were conducted under closed-loop adaptive control with a 1 second sampling period, where each execution spans approximately 8.5 hours. The files capture diverse operational conditions, including steady-state baselines and disturbance scenarios with abrupt outflow variations. Each dataset contains timestamps, valve positions, tank levels, control signals, tracking errors, adaptive PID gains tuned via Dahlin’s synthesis, and real-time model parameters estimated using recursive least squares. Data quality assessment confirms the integrity of the collection through exploratory analysis, controller behavior evaluation across multiple regimes, and a baseline anomaly detection case study. Capturing key nonlinearities such as turbulence and actuator saturation, this resource primarily supports control engineering tasks including adaptive control evaluation, disturbance rejection analysis, and data-driven monitoring, while also providing a testbed for control-oriented identification under realistic closed-loop conditions.

## Background & Summary

Modern approaches to system identification and automatic control increasingly rely on data collected from physical systems. As sensing, computation, and connectivity continue to advance, data-driven modeling techniques and adaptive control play a critical role in industrial, infrastructure, and cyber-physical applications^1,2^. These methods require datasets that adequately represent the dynamics, nonlinearities, and operational variability of real processes. However, many engineering studies still rely heavily on simulations or simplified experimental setups, which often fail to capture critical physical phenomena such as turbulence, actuator limits, long-term drift, and measurement noise^3,4^.

This reliance on simulation stems from the fact that public datasets in process control remain limited, particularly for nonlinear systems operating under diverse conditions. Existing collections often focus on nominal regimes, steady-state operation, or short experimental durations, restricting their usefulness for evaluating identification and control strategies that must operate reliably across dynamic and disturbed scenarios^2,3,5^. Furthermore, adaptive control algorithms that update their parameters based on real-time data are susceptible to issues that are difficult to simulate faithfully, including sensor degradation, hydraulic nonlinearities, and abrupt disturbances. In this context, datasets that capture real disturbances, nonlinear flow effects, and evolving identified parameters are particularly valuable to the research community.

These challenges highlight the importance of experimental platforms capable of naturally producing such nonlinearities and parameter variations. Among the systems commonly used for this purpose, the two tank water system stands out as a well known benchmark in automatic control research due to its nonlinear hydraulic behavior, ease of physical implementation, and rich dynamics despite its conceptual simplicity^6^. Given the need for benchmarks that bridge the gap between theory and practice, this system represents an ideal testbed for generating high fidelity control data.

To address these limitations, here we present an openly accessible experimental dataset collected from a laboratory scale two tank water pumping plant. The collection comprises 25 long-duration time-series experiments conducted under three operational conditions: Steady-State Baseline (Minimal Outflow), Steady-State with Passive Outflow, and Dynamic Disturbance Rejection. In this system, passive outflow refers to the gravity driven drainage imposed by a manual valve, which defines the uncontrolled baseline leakage of the upper tank. Each experiment includes process measurements, control signals, setpoints, and real-time estimates of system parameters obtained via recursive least squares (RLS), together with the corresponding adaptive PID gains updated through Dahlin’s tuning method. This combination of variables provides a unique opportunity to study interactions between physical dynamics, system identification, and adaptive control under realistic conditions.

The goal of making these datasets publicly available is to support reproducible research and provide a structured benchmark for evaluating adaptive control strategies, data-driven monitoring algorithms, and control-oriented identification methods. It is important to note that the experimental design prioritizes the exploration of diverse operating regimes and the evaluation of adaptive controller behavior over the generation of persistently exciting signals typically required for high-precision offline system identification. This reflects a deliberate trade-off: closed-loop operation inherently reduces input excitation, but it enables the capture of realistic controller–plant interactions that are difficult to replicate in simulation. Consequently, the dataset is particularly well-suited for assessing adaptive control performance, disturbance rejection capabilities, and anomaly detection algorithms, while its utility for pure system identification may be limited to control-oriented or low-frequency model approximations. The availability of multi-condition experimental data enables researchers and practitioners to assess algorithmic performance across operating regimes that are challenging to replicate in simulation.

## Methods

This section details the experimental design, encompassing the physical plant configuration, the data acquisition architecture, and the specific protocols used to generate the dataset. The following subsections provide comprehensive specifications of the hardware components and the control algorithms to facilitate the correct interpretation of the recorded variables and ensure the reproducibility of the study.

### Experimental Setup

**Fig. 1:**
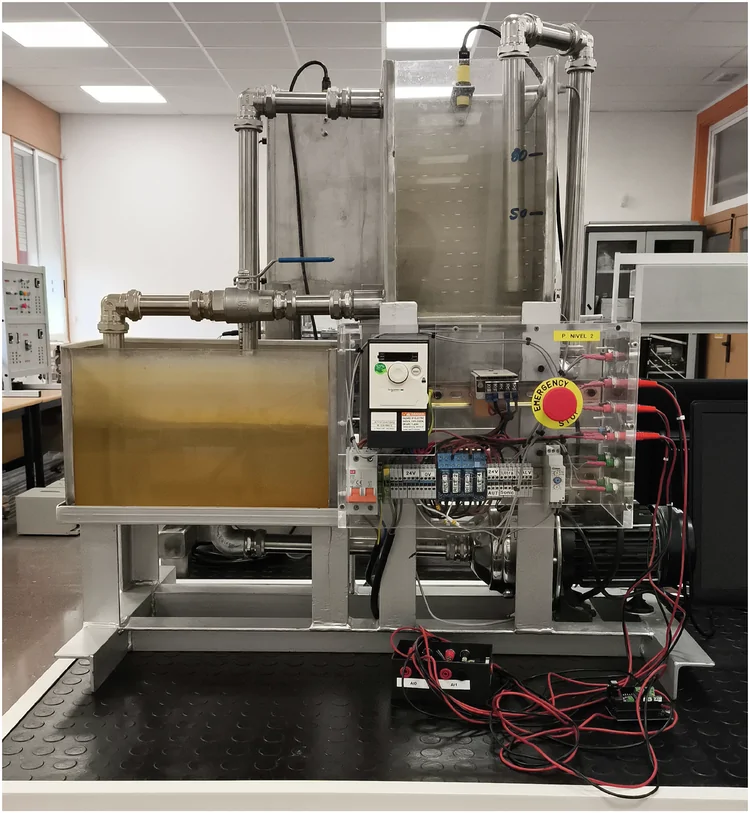
Photograph of the laboratory-scale two-tank plant at EPEF.

**Fig. 2:**
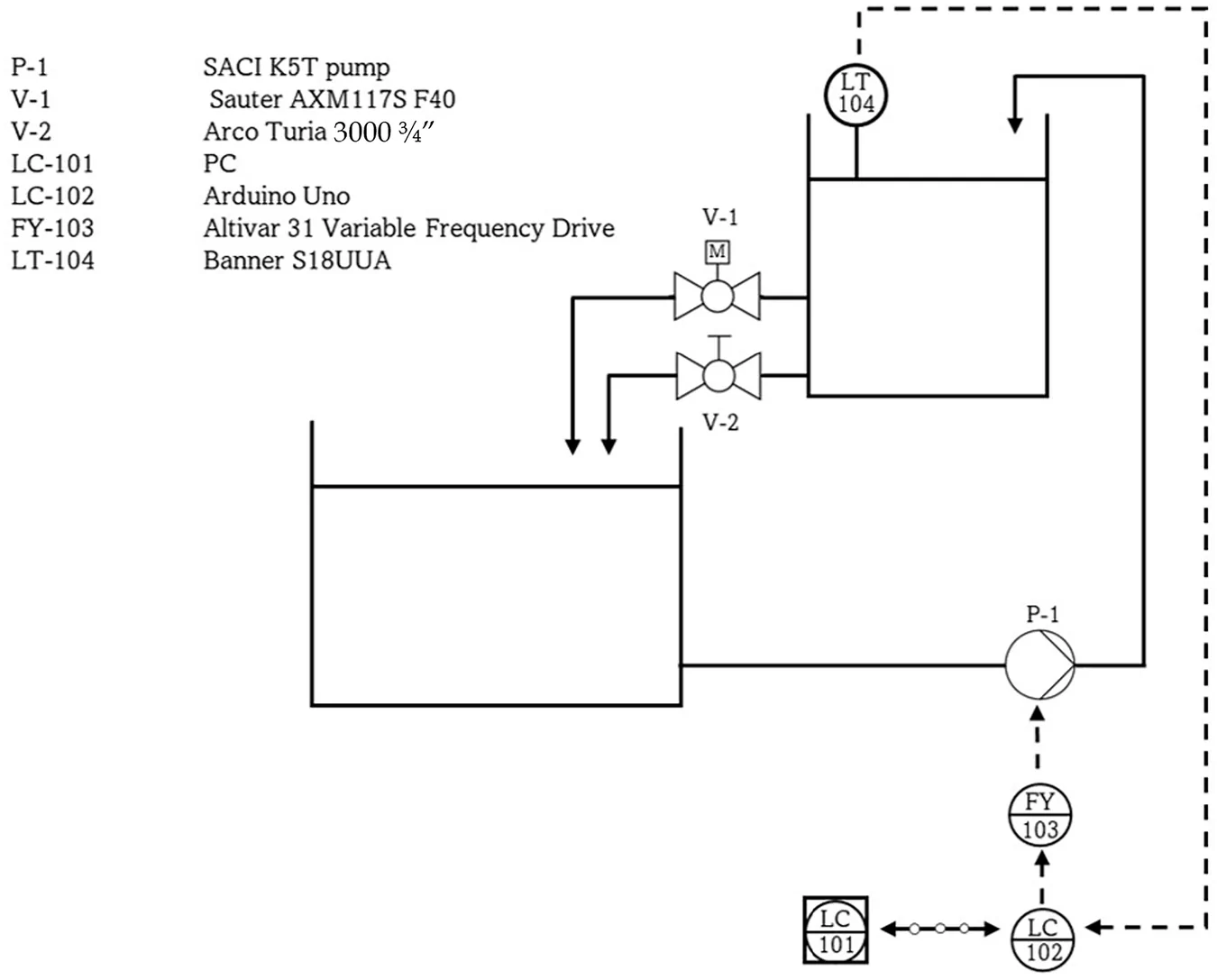
Schematic of the experimental setup.

The experimental data were acquired from a laboratory scale two tank water pumping system, which can be seen in Fig. 1, located at the Escola Politécnica de Enxeñariá de Ferrol (EPEF), Universidade da Coruña. The plant consists of an upper tank, where the water level is controlled, and a lower reservoir tank that acts as a water supply with effectively infinite capacity relative to the upper tank dynamics. Only the upper tank level is measured and controlled; the lower reservoir serves exclusively as a buffer, maintaining a constant supply without contributing measurable dynamics to the system. From a system identification perspective, this configuration corresponds to a single-input single-output (SISO) system with one measured dynamic state: the upper tank level. The upper tank has internal dimensions of 32 × 21.5 × 21.5 cm, while the lower reservoir measures 22 × 35 × 25 cm. Water is pumped from the lower tank to the upper tank and returns through two discharge pipes; all the pipes in the system are metallic with a 3/4" internal diameter. A schematic representation of the setup is shown in Fig. 2.

The main hardware components are:

- **Inflow System:** The inflow to the upper tank is provided by a SACI K5T centrifugal pump driven by an Altivar 312 Variable Frequency Drive (VFD) (model ATV312H075M2, Schneider Electric). This VFD receives a 230 V single-phase AC input and generates a three-phase output whose voltage and frequency are modulated proportionally by a 0–10 V DC control signal. This configuration enables smooth and continuous adjustment of the pump speed throughout the experiments. In addition, the VFD includes an auxiliary 24 V DC output that can power external devices, such as the data acquisition interface or other low power instrumentation.
- **Level Sensor:** The water level in the upper tank is measured using a Banner S18UUA ultrasonic sensor (Banner Engineering Corp.). The sensor operates over a 30–300 mm detection range, covering the full 0–25 cm working height of the tank. It provides a 0–10 V analog output proportional to the water column. According to the manufacturer’s specifications^7^, the sensor has a resolution of 0.5 mm and a linearity of  ± 0.5 mm (at 30 ms response time), with a temperature effect of 0.02% of distance per ^∘^C. These specifications apply to measurements using a 50 mm  × 50 mm aluminum plate under fixed sensing conditions; under turbulent hydraulic conditions with an oscillating water surface, the effective measurement uncertainty increases due to surface fluctuations and ultrasonic beam scattering. This voltage is linearly downscaled to the input range of the acquisition electronics through an external signal conditioning stage.
- **Outflow System:** Two discharge pipes enable manipulation of the outflow, one incorporates a fully manual valve (Arco Turia 3000, 3/4”) and the other a controllable electrovalve (Sauter AXM117S F40). These two valves allow the creation of a wide variety of hydraulic conditions, from nearly no leakage to rapid drainage with strong turbulence.

This configuration reproduces key nonlinearities characteristic of hydraulic processes, such as turbulent flow under high drainage conditions, nonlinear level–flow relationships, and actuator constraints inherent to VFD driven pump.

### Experimental Procedure

A total of 25 experiments were performed following a structured protocol designed to expose the system to a variety of hydraulic regimes and dynamic conditions. Several categories of experiments were conducted: steady-state operation under different outflow configurations and disturbance rejection experiments with abrupt changes in discharge conditions.

#### Steady-State Experiments

Twenty one experiments were performed under steady-state conditions to characterize the plant response over different fixed operating points. Each experiment varies the opening of the controllable electrovalve while the manual discharge valve imposes one of two distinct passive outflow regimes.

- **With Constant Passive Outflow:** In these 11 experiments, the manual discharge valve was kept fully open (100%), creating a constant passive outflow. The controllable electrovalve’s opening was then set to a fixed percentage, from 0% to 100% in 10% increments. It should be noted that this experimental configuration, with the manual discharge valve fully open, often resulted in turbulent water flow within the tank, which influenced the quality of the sensor measurements.
- **With Minimal Passive Outflow (Baseline):** Ten baseline experiments were conducted with the manual valve fully closed, eliminating passive leakage. The controllable electrovalve was again set to fixed openings ranging from 10% to 100%. This configuration provides a smoother hydraulic response with minimal disturbance from uncontrolled drainage, serving as a reference regime for comparison.

#### Disturbance Rejection Experiments

Four additional experiments were designed to evaluate the controller’s ability to reject external disturbances arising from abrupt outflow changes. Each disturbance lasted approximately one hour and involved a sudden modification of the manual valve aperture.

- **High-Outflow Disturbance:** Two experiments were conducted with the controllable electrovalve set to high fixed openings (80% and 90%) and the manual valve initially fully open. The disturbance was introduced by closing the manual valve to approximately 50%, reducing the outflow and altering the tank’s hydraulic behavior.
- **Low-Outflow Disturbance:** Two additional experiments were conducted with the controllable valve at 50% and 60% while the manual valve remained fully closed. The disturbance consisted of abruptly opening the manual valve to approximately 50%, resulting in a rapid and nonlinear increase in drainage.

In all experiments, the controller tracked a staircase reference profile starting at 10% and increasing in steps of 5% up to 90%, each maintained for 30 minutes. Each full run lasted approximately 8.5 hours.

### Data Acquisition and Control Algorithm

The data acquisition and control architecture integrates the physical instrumentation of the two tank system with a MATLAB based supervisory computer and an embedded Arduino interface. This subsystem is responsible for sampling the level sensor, conditioning and converting analog signals, executing the adaptive control law, and actuating the pump and the controllable valve with deterministic timing.

#### Data Acquisition Architecture

**Fig. 3:**
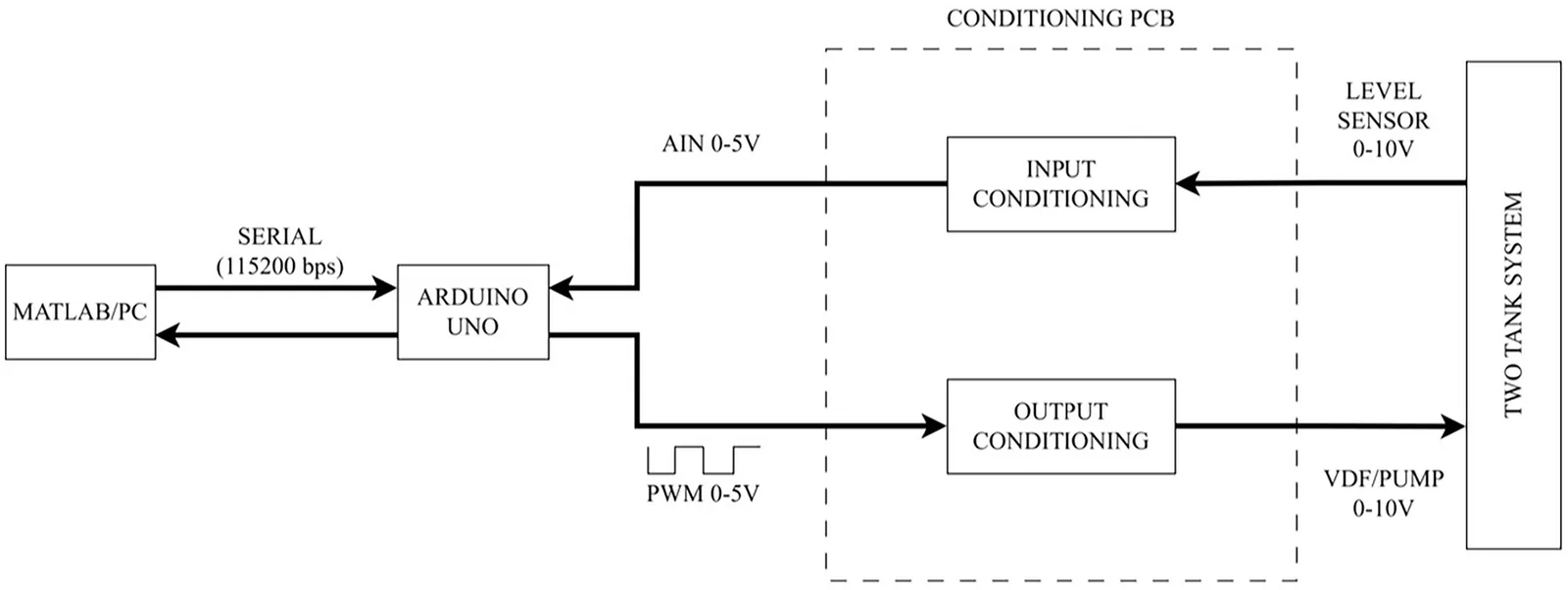
Diagram of the control and data acquisition system.

The specific hardware implementation focuses on signal conditioning and electrical compatibility to ensure accurate data exchange. As illustrated in Fig. 3, a dedicated interface circuit bridges the operational power range of the industrial instrumentation with the logic levels of the embedded microcontroller. This configuration enables the synchronized acquisition and actuation sequence described below.

The water level is measured using the Banner S18UUA, which provides a 0–10 V analog output proportional to the 0–25 cm water column. This voltage is downscaled to the 0–5 V range required by the Arduino ADC using the external signal-conditioning PCB. The Arduino generates a PWM control signal, which is filtered to produce a smooth 0–10 V analog command, using the same conditioning circuit mentioned before. This drives the VFD, which receives 230 V AC single phase and supplies a three phase V/Hz controlled output to the SACI K5T pump. The controllable discharge path, however, is actuated by an external voltage generator.

MATLAB communicates with the Arduino via a 115200 bps serial link. Each acquisition–control cycle is executed with a 1 s sampling period. During each iteration, MATLAB requests the most recent level measurement, computes the control signal, transmits the updated actuation command, and logs all relevant variables, including timestamps, measurements, actuator states, and model parameters.

This architecture ensures synchronized sampling, deterministic loop execution, and tight coupling between the physical plant and the control algorithms described in the following subsections.

#### Real-time System Identification

A Recursive Least Squares (RLS) algorithm with a forgetting factor of *λ*_*RLS*_ = 0.9 was implemented to identify the dynamics of the two tank system in real-time. The plant was modeled as a second order discrete time transfer function with a one sample time delay, a common structure in system identification^8^:

1 $$G(z)=\frac{Y(z)}{U(z)}=\frac{{b}_{1}{z}^{-1}}{1+{a}_{1}{z}^{-1}+{a}_{2}{z}^{-2}}$$

where *Y*(*z*) is the system output (*Level_pct*), *U*(*z*) is the system input (*Control_Signal_pct*), and *z*^−1^ is the backward shift operator. The parameter vector to be estimated is $\theta ={[{a}_{1},{a}_{2},{b}_{1}]}^{T}$.

The choice of this second-order model structure represents a control-oriented approximation rather than a physically derived model. While a first-order model would be the minimal representation for a single-tank level dynamics, a second-order structure was selected to provide the RLS algorithm with additional degrees of freedom to capture unmodeled dynamics, such as pump response delays, sensor filtering effects, and hydraulic transients. This pragmatic choice prioritizes controller tuning flexibility over physical interpretability of the identified parameters.

At each time step *k*, the algorithm updates the parameter estimate $\widehat{\theta }(k)$ based on the new measurement *y*(*k*) and the regression vector *ϕ*(*k*) = [−*y*(*k*−1), −*y*(*k*−2), *u*(*k*−1)]^*T*^. The standard RLS update equations are as follows:

2 $$e(k)=y(k)-\phi {(k)}^{T}\widehat{\theta }(k-1)$$

3 $$K(k)=\frac{P(k-1)\phi (k)}{{\lambda }_{RLS}+\phi {(k)}^{T}P(k-1)\phi (k)}$$

4 $$\widehat{\theta }(k)=\widehat{\theta }(k-1)+K(k)e(k)$$

5 $$P(k)=\frac{1}{{\lambda }_{RLS}}\left[P(k-1)-K(k)\phi {(k)}^{T}P(k-1)\right]$$

Here, *e*(*k*) represents the a priori prediction error, *K*(*k*) is the estimator gain vector, and *P*(*k*) denotes the covariance matrix of the estimation error.

For the algorithm’s initialization, the parameter estimates in $\widehat{\theta }(0)$ were seeded with arbitrary random values. To reflect the high degree of uncertainty associated with these initial guesses, the covariance matrix, *P*(0), was initialized with large values on its diagonal. This standard practice ensures that the initial parameter estimates can converge rapidly. The RLS update began at the second time step (*k* = 2), once a full regression vector was available. The estimated parameters were then passed to the controller tuning stage.

#### Adaptive PID Controller Synthesis

A self-tuning Proportional-Integral-Derivative (PID) controller was implemented to regulate the water level. The general form of the discrete PID controller used is based on established theory^9^:

6 $${G}_{c}(z)={K}_{p}+\frac{{K}_{i}{T}_{s}}{1-{z}^{-1}}+\frac{{K}_{d}}{{T}_{s}}(1-{z}^{-1})$$

The gains *K*_*i*_ and *K*_*d*_ used are derived from the standard integral time (*T*_*i*_) and derivative time (*T*_*d*_) as follows:

7 $${K}_{i}=\frac{{K}_{p}}{{T}_{i}}$$

8 $${K}_{d}={K}_{p}\cdot {T}_{d}$$

The values of the controller’s gains were continuously updated based on the identified model parameters using Dahlin’s synthesis ^10^. The tuning is governed by a single design parameter, *λ*, which was set to a constant value of *λ* = 3.0 for all experiments to achieve a balance between response speed and stability. At each time step, the PID parameters were calculated from the identified model and the parameter *λ* according to the following synthesis equations:

9 $$Q=1-{e}^{-{T}_{s}/\lambda }$$

10 $${K}_{p}=-\frac{({a}_{1}+2{a}_{2})Q}{{b}_{1}}$$

11 $${T}_{d}=\frac{{T}_{s}\cdot {a}_{2}\cdot Q}{{K}_{p}\cdot {b}_{1}}$$

12 $${T}_{i}=-\frac{{T}_{s}}{\frac{1}{{a}_{1}+2{a}_{2}}+1+\frac{{T}_{d}}{{T}_{s}}}$$

where *T*_*s*_ represents the sampling period (1s), *a*_1_, *a*_2_, *b*_1_ are the model parameters estimated by the RLS algorithm, and *Q* is an auxiliary variable determined by the tuning parameter *λ*.

**Fig. 4:**
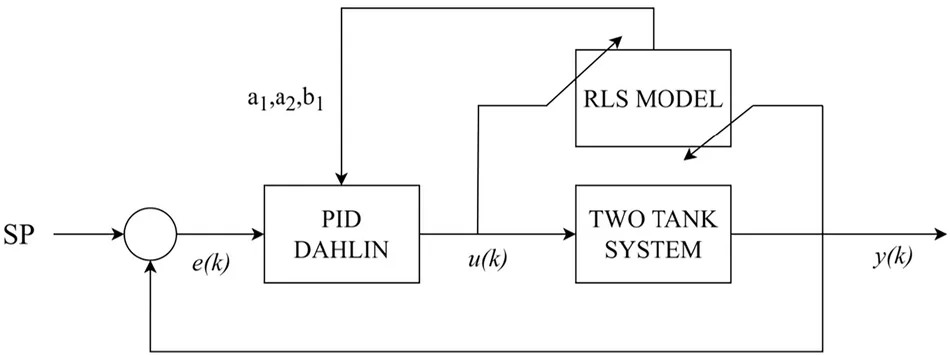
Diagram of the adaptive control process.

The control signal, *u*(*k*), was calculated using the incremental form of the PID algorithm and was saturated to a range of [0, 100] to respect the physical limits of the pump’s VFD. Figure 4 shows the resultant diagram of the adaptive control process.

## Data Record

The complete dataset is publicly available in the Zenodo repository^11^. The collection consists of 765000 samples divided into 25 comma-separated value (CSV) files, each corresponding to a single long-duration experiment of approximately 8.5 hours. All files share a fixed sampling frequency of 1 Hz and contain either 12 or 13 columns, depending on the experiment type. The ones done for disturbance rejection include an additional column indicating the presence of an externally applied outflow perturbation. The experiments are grouped into three subsets that reflect different hydraulic operating conditions. All data files include timestamped records of process measurements, control actions, identified model parameters, and adaptive PID gains. Each CSV file follows the following naming structure:

- Baseline_Xpct.csv for steady-state experiments with minimal passive outflow
- PassiveOutflow_Xpct.csv for steady-state experiments with constant passive outflow
- Disturbance_Condition_Xpct.csv for dynamic disturbance rejection experiments

In this structure, X indicates the closure percentage of the controllable electrovalve, and Condition refers to the disturbance scenario (HighOutflow or LowOutflow).

Table 1 provides a concise summary of all 25 experiments, including the operating conditions, valve configurations, and approximate sample counts.

**Table 1:** Summary of experimental conditions.

Category	Condition	Manual Valve	Electrovalve Range	Experiments
Baseline	Minimal outflow	Closed (0%)	10%–100%	10
Passive Outflow	Constant outflow	Open (100%)	0%–100%	11
Disturbance (High)	Outflow decrease	100% → 50%	80%, 90%	2
Disturbance (Low)	Outflow increase	0% → 50%	50%, 60%	2
**Total experiments**				**25**
**Total samples**				** ≈ 765,000**
**Duration per experiment**				** ≈ 8.5 hours**
**Sampling period**				**1 second**

**Fig. 5:**
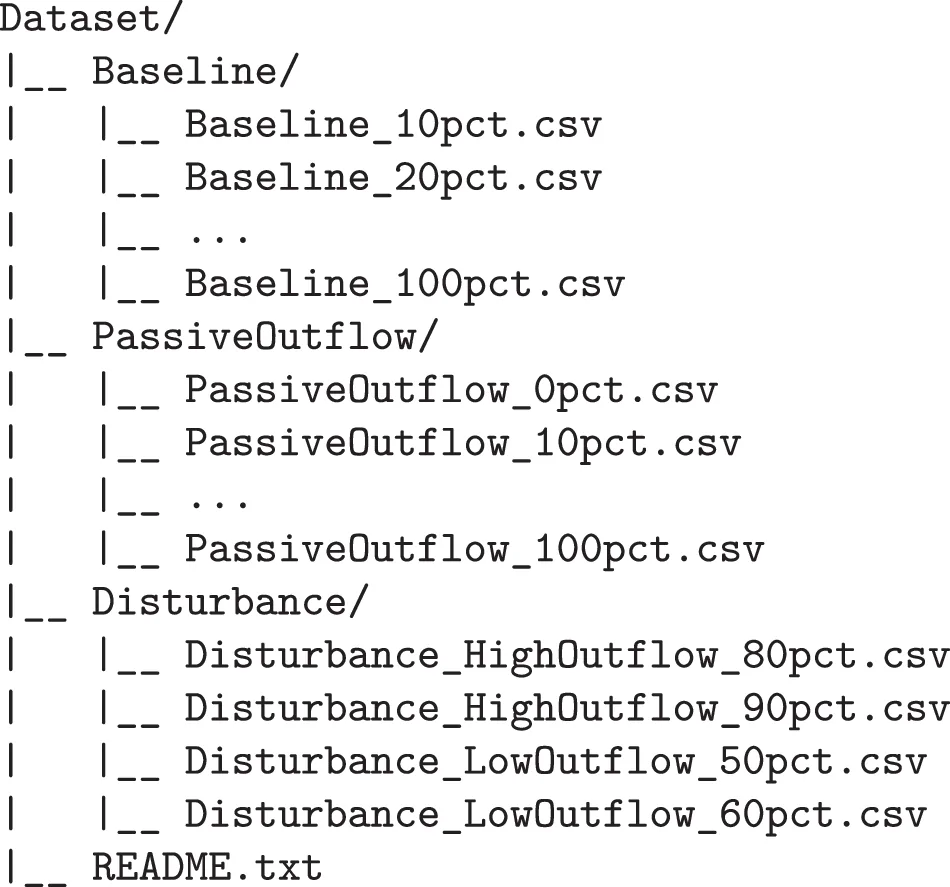
Directory structure of the dataset repository.

All files use UTF-8 encoding and are stored with Unix-style line endings. Numeric values use a dot as the decimal separator. The directory structure of the dataset repository is shown in Fig. 5. Table 2 summarizes the dataset columns (variables), their physical meaning, units and data type. All signals are recorded without post-processing to preserve the raw dynamics of the system. Variables expressed in percentage (%) are normalized to their respective full-scale ranges: 0–25 cm for water level, 0–10 V control voltage (corresponding to 0–50 Hz pump frequency), and 0–100% valve aperture.

**Table 2:** Description of dataset variables.

Variable	Description	Unit	Type
Timestamp	Format (YYYY-MM-DD HH:MM:SS)	—	string
Valve_Closure_pct	Electrovalve commanded closure	%	float
Level_pct	Measured water level in upper tank	%	float
Control_Signal_pct	Pump actuation signal (VFD, scaled)	%	float
Setpoint_pct	Level reference command	%	float
Error_pct	Tracking error (Setpoint − Level)	%	float
Kp	Adaptive proportional gain	—	float
Ki	Adaptive integral gain	—	float
Kd	Adaptive derivative gain	—	float
b1	RLS-identified process gain	—	float
a1	RLS-identified first parameter	—	float
a2	RLS-identified second parameter	—	float
Perturbation	Binary indicator of disturbance^1^	—	boolean

## Technical Validation

To ensure the reliability and integrity of the dataset, a comprehensive Exploratory Data Analysis (EDA) was performed across the three experimental categories. This statistical assessment was designed to confirm the consistency of the closed-loop control, verify the capture of physical nonlinearities, such as actuator saturation and turbulence, and characterize the behavior of the adaptive algorithms. The analysis is structured into three parts corresponding to the operational regimes, followed by observations on the physical interpretation and a specific case study on anomaly detection. It should be noted that this section focuses on data quality and consistency rather than formal validation of identification accuracy, which would require dedicated experiments with persistently exciting inputs.

### Analysis of Baseline Experiments

The baseline experiments with minimal passive outflow serve as the reference for nominal system operation. This analysis validates the controller’s performance and the physical consistency of the data under stable conditions.

#### Tracking Performance and Coverage

**Fig. 6:**
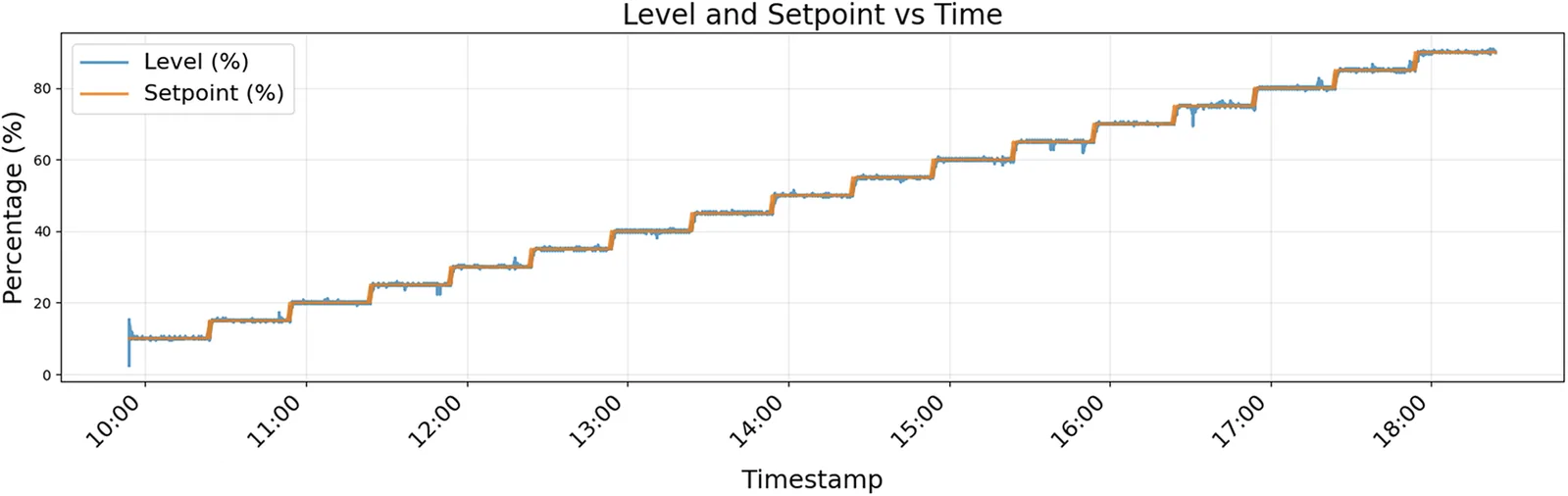
Example of the level behavior in the Steady-State Baseline Experiment (Minimal Passive Outflow).

Figure 6 demonstrates the fulfillment of the primary control goal: the process variable (Level_pct) successfully tracks the step-wise Setpoint_pct profile. As quantified in Table 3, the system achieves high precision with a mean tracking error of only 0.059% and a standard deviation of 0.69%.

**Table 3:** Descriptive Statistics for Process Variables (Baseline).

	Level	Control Sig.	Setpoint	Error	Valve Clos.
**Count**	305989	305989	305989	305989	305989
**Mean (%)**	49.94	16.93	50.00	0.059	45.00
**Std (%)**	24.46	4.87	24.49	0.69	28.72
**Min (%)**	0.39	0.00	10.00	-22.15	0.00
**Max (%)**	99.60	100.00	90.00	9.60	90.00

^1^This variable is present only in the disturbance rejection datasets.

**Fig. 7:**
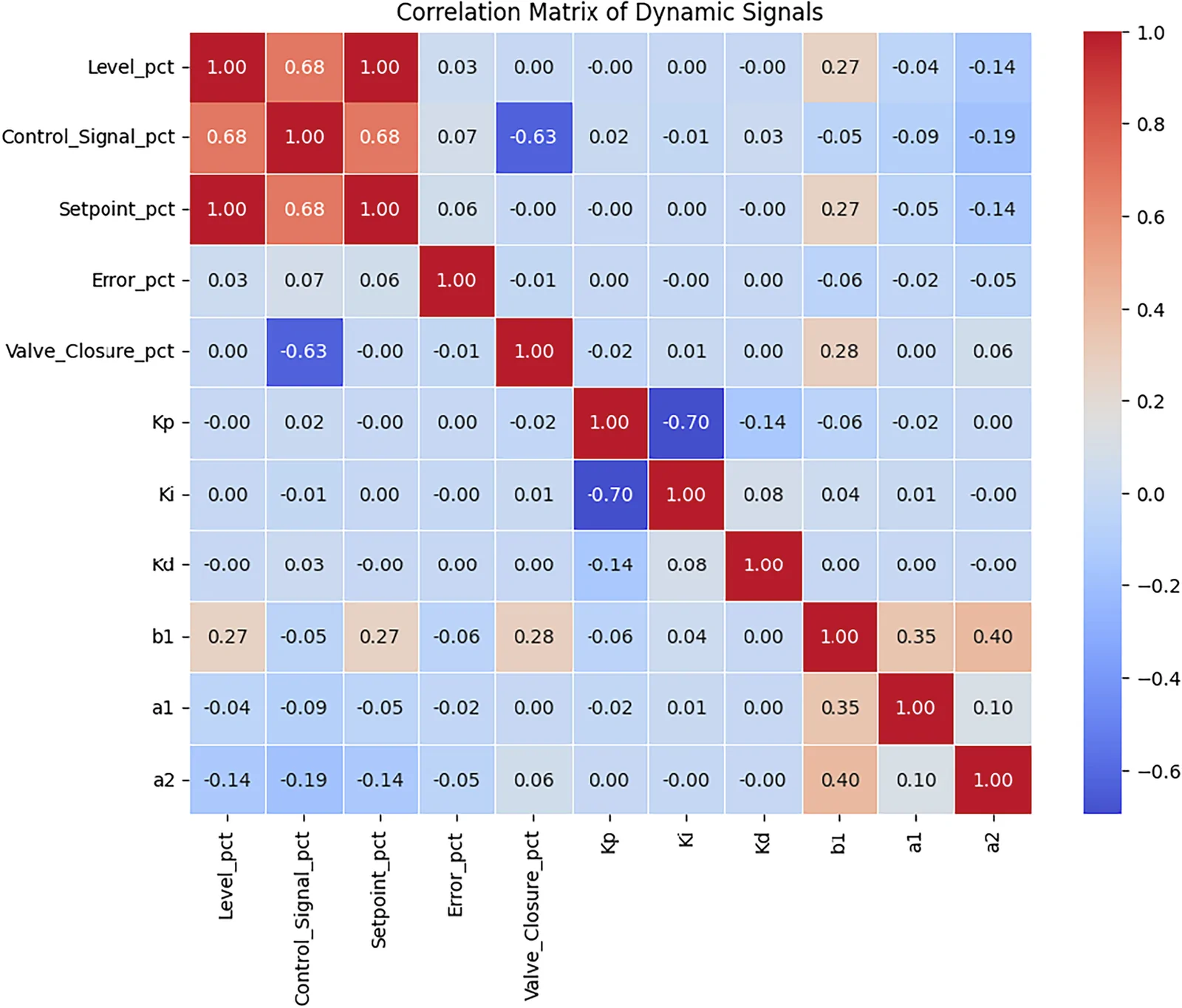
Correlation matrix of dynamic signals for the Baseline experiments.

**Fig. 8:**
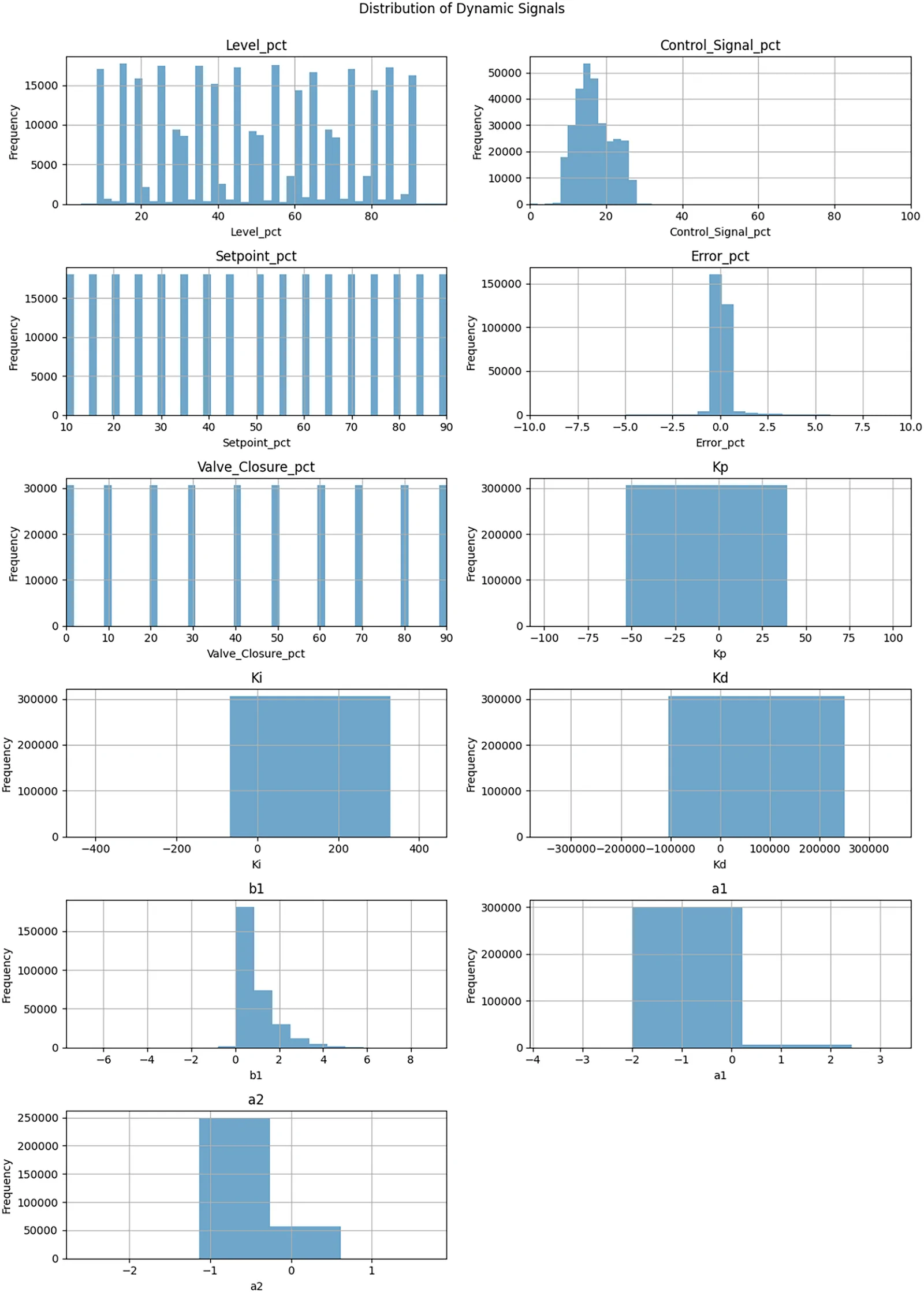
Distribution of dynamic signals collected during the Baseline experiments.

The histograms in Fig. 8 confirm the success of the experimental design. The near-uniform distributions for Setpoint_pct and Valve_Closure_pct indicate that the experiments captured data equitably across the entire operating range (10%–90%), as intended. In sharp contrast, the Error_pct distribution is tightly concentrated at zero, visually confirming the controller’s performance.

#### Validation of System Dynamics and Adaptation

The correlation matrix (Fig. 7) reveals key physical relationships that validate the dataset integrity:

- A strong positive correlation between Control_Signal_pct and Setpoint_pct, consistent with the physics of pumping systems (higher levels require more energy).
- A negative correlation between Valve_Closure_pct and Control_Signal_pct, correctly reflecting that restricting the outflow reduces the pump effort required to maintain the level.
- The independence of Error_pct from other variables, indicating that deviations are primarily due to random noise rather than systematic control failures.

Regarding the adaptive mechanism, the data in Table 4 demonstrate that the RLS algorithm was continuously active. The variability in the estimated parameters (particularly Ki and Kd) proves that the algorithm was identifying dynamic changes in real-time and updating the controller gains via Dahlin’s synthesis.

**Table 4:** Descriptive Statistics for Model Parameters (Baseline).

	Kp	Ki	Kd	b1	a1	a2
**Count**	305989	305989	305989	305989	305989	305989
**Mean**	0.86	−2.36	−4.13e1	0.96	−0.22	−0.42
**Std**	10.91	47.04	3.82e4	0.86	0.38	0.23
**Min**	−2280	−6841	−1.21e7	−33.17	−103.3	−33.58
**Max**	2358	13079	5.55e6	8.34	6.83	10.26

### Analysis of Steady-State Experiments With Constant Passive Outflow

This set of experiments validates the richness of the dataset by capturing the system’s behavior under challenging operational conditions. To ensure a clear analysis, samples where the level registered as zero (anomalies caused by excessive turbulence interfering with sensor readings) were removed during post-processing. The data in this section do not demonstrate perfect control; instead, they validate the capture of two critical real-world phenomena: actuator saturation and sensor noise.

#### Validation of Physical Limitations (Saturation)

**Fig. 9:**
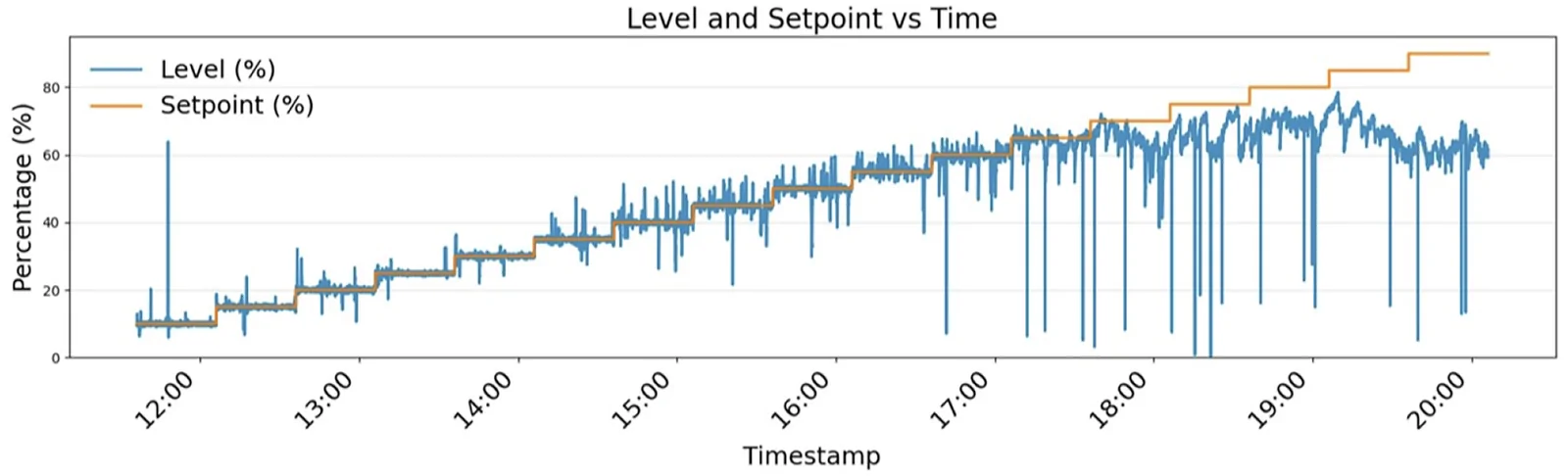
Level behavior in the Steady-State Experiment with Constant Passive Outflow, showing saturation effects.

The control objective is not fully met in this scenario, providing valuable failure data. As shown in Fig. 9, there is a clear tracking failure: while the Level_pct follows the setpoint at low levels, it systematically fails to reach targets above  ≈ 65%.

**Fig. 10:**
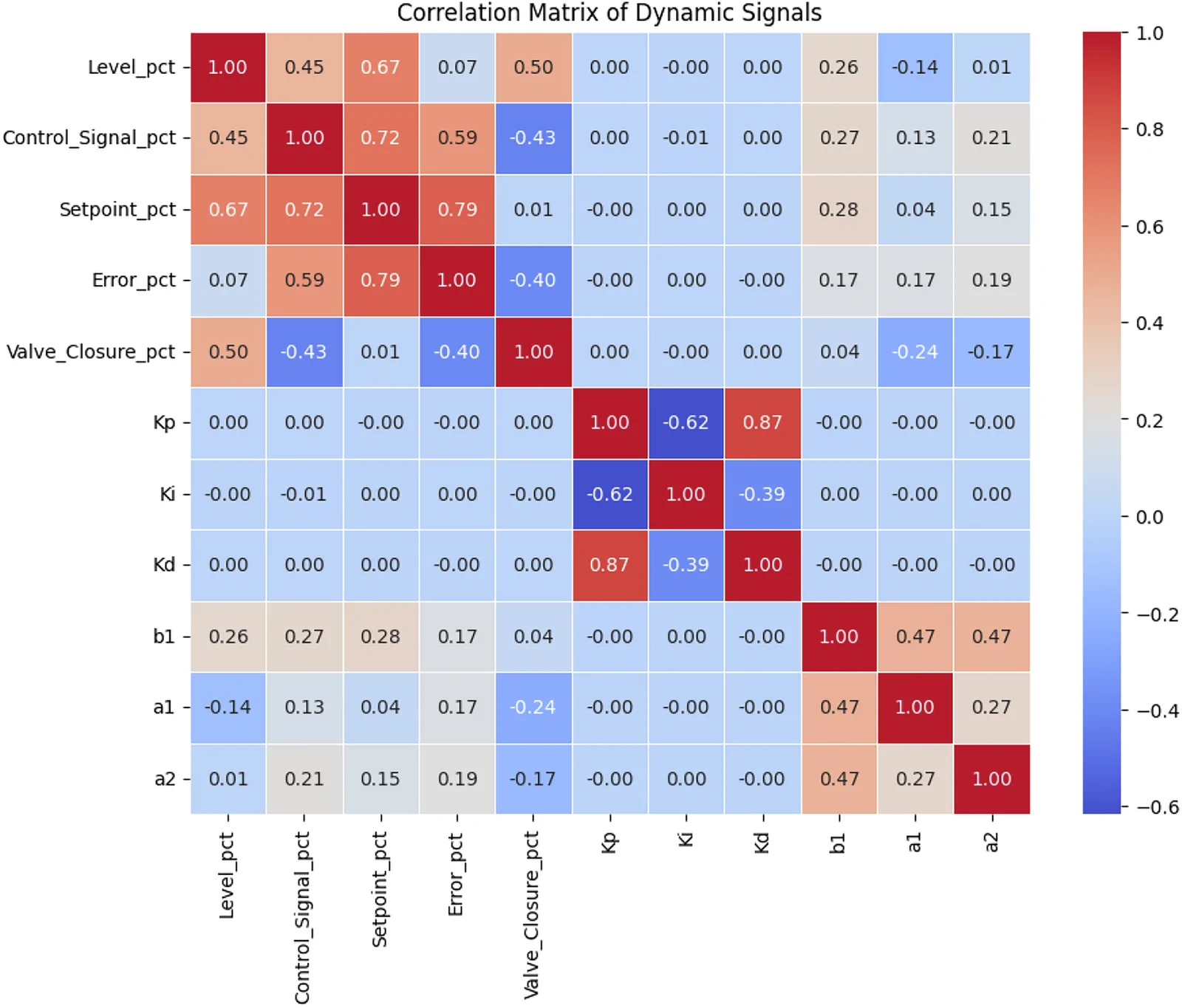
Correlation matrix of dynamic signals for the Constant Passive Outflow experiments.

**Fig. 11:**
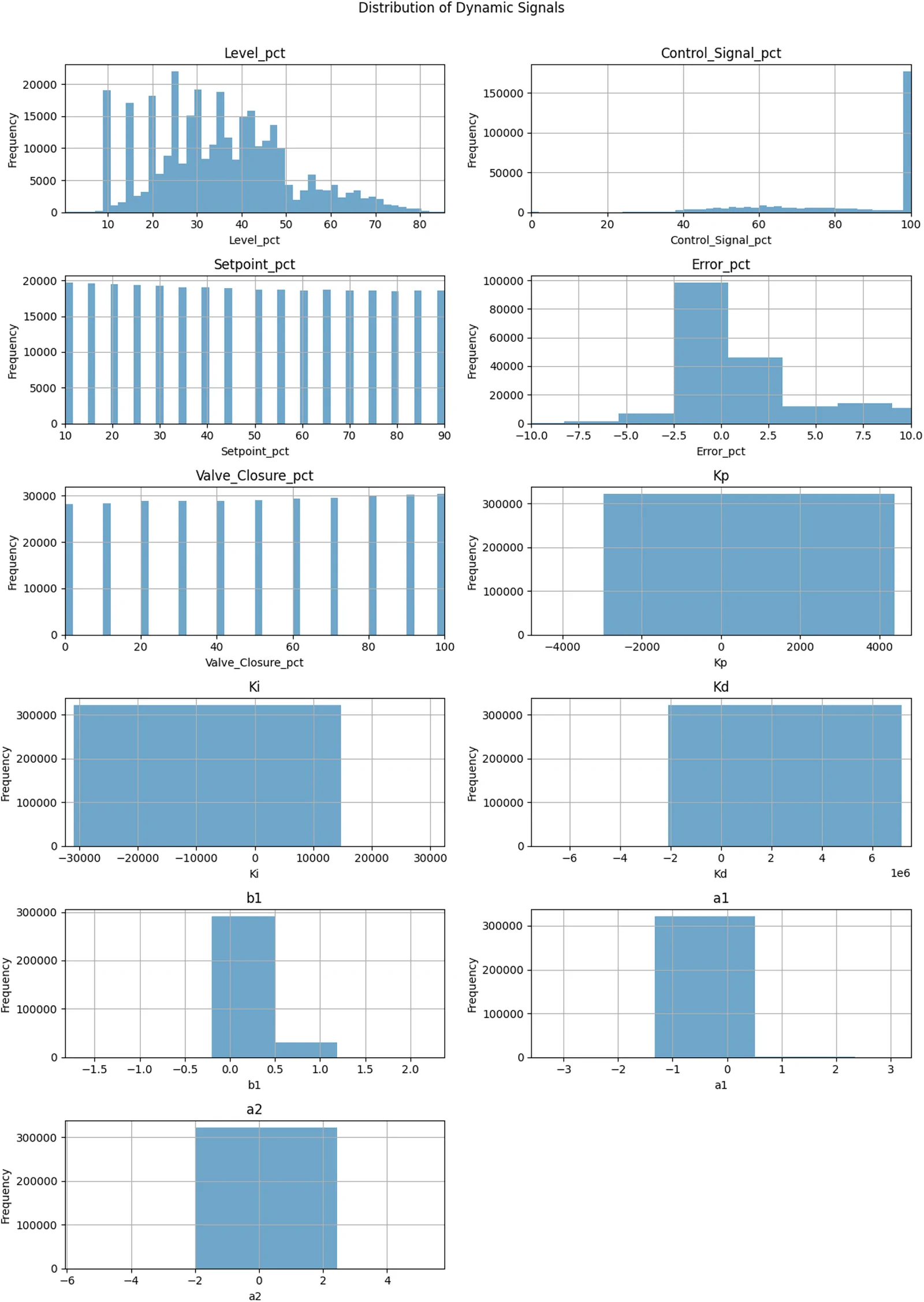
Distribution of dynamic signals for the Constant Passive Outflow experiments.

The cause is physically identified in the statistics of Table 5. The mean control signal is 84.47%, and the distribution in Fig. 11 shows a massive concentration at the 100% maximum. This proves the controller was operating in saturation, physically incapable of overcoming the high passive outflow. This validates the dataset as an excellent testbed for control algorithms that must handle anti-windup and physical constraints.

**Table 5:** Descriptive Statistics for Process Variables (Constant Outflow).

	Level	Control Sig.	Setpoint	Error	Valve Clos.
**Count**	321505	321505	321505	321505	321505
**Mean (%)**	35.06	84.47	49.52	14.45	41.61
**Std (%)**	15.20	20.35	24.55	18.30	30.33
**Min (%)**	0.39	0.00	10.00	−54.31	0.00
**Max (%)**	85.49	100.00	90.00	89.60	90.00

#### Validation of Sensor Noise and Adaptive Response

The high outflow generated significant turbulence. Consequently, the Level_pct trace in Fig. 9 appears as an erratic band rather than a smooth line. This high-frequency noise validates the dataset’s authenticity by including non-ideal measurement artifacts typical of ultrasonic sensors in fluids.

Regarding the adaptive algorithm, noise and saturation create a challenging environment for identification. Table 6 reveals extreme variance and outliers in the estimated parameters. This volatility reflects the response of the RLS algorithm under saturated, nonlinear conditions. The Correlation Matrix (Fig. 10) shows that the structural relationships between the Dahlin synthesis gains (Kp, Ki, Kd) are preserved.

**Table 6:** Descriptive Statistics for Model Parameters (Constant Outflow).

	Kp	Ki	Kd	b1	a1	a2
**Count**	321505	321505	321505	321505	321505	321505
**Mean**	1.95	−3.25	1.19e3	0.27	−0.10	−0.13
**Std**	479.4	3.24e3	7.53e5	0.21	0.34	0.59
**Min**	−1.4e5	−1.17e6	−3.9e7	−24.5	−65.5	−191.5
**Max**	2.2e5	1.11e6	4.2e8	10.2	26.2	28.8

### Analysis of Disturbance Rejection (Outflow Increase)

These experiments validate the successful capture of a high-impact, transient disturbance event. The analysis confirms that the aggregated statistics, combined with the time-series visualization, provide a coherent picture of the system’s fault response.

#### Validation of Transient Response and Event Signature

**Fig. 12:**
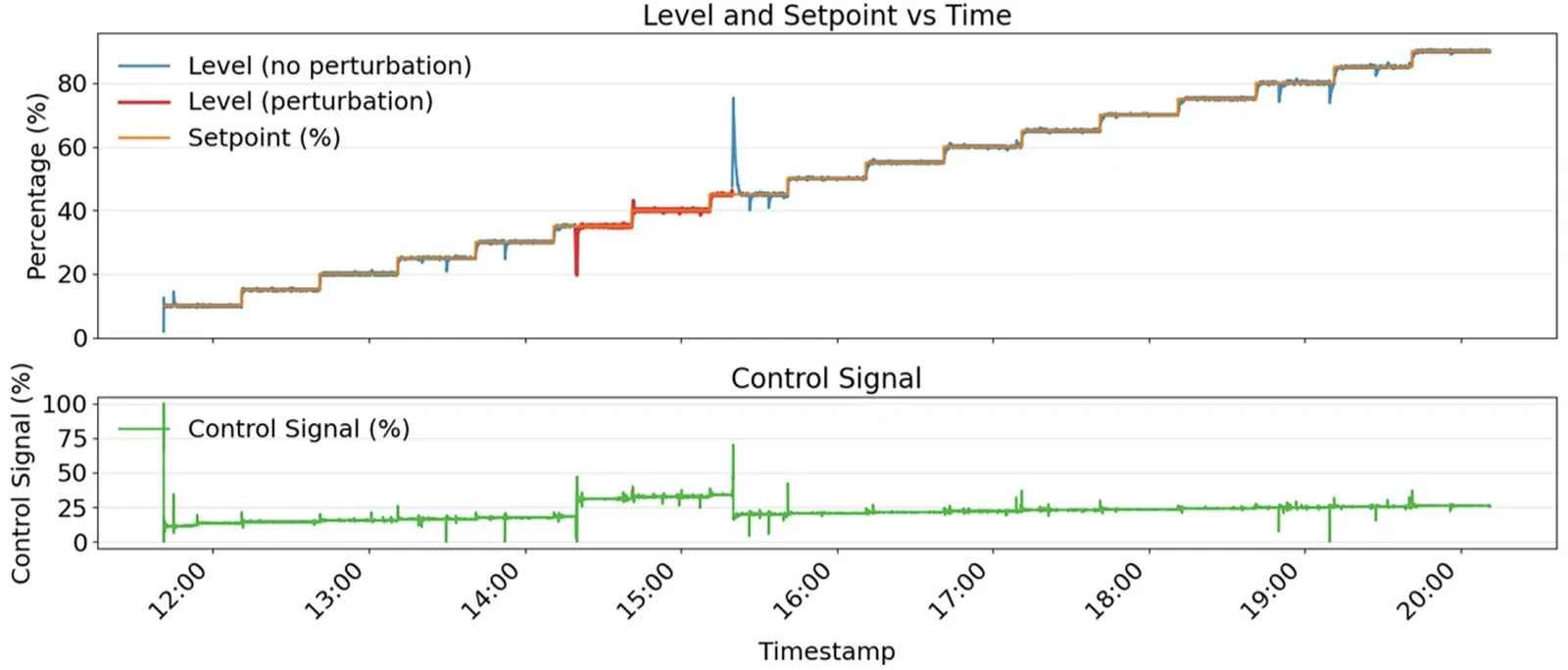
Time-series response during the Outflow Increase Disturbance, showing the level drop and controller recovery.

Figure 12 provides the primary visual evidence. The top plot captures the disturbance dynamics: the sudden manual valve operation causes the Level_pct to drop, creating a large positive tracking error, followed by a recovery phase with a characteristic overshoot (negative error).

Table 7 quantifies these events. While the overall mean error remains low (0.03%), the event severity is validated by the extrema:

- **Max Error (15.39%):** Quantifies the magnitude of the level drop.
- **Min Error(−30.29%):** Quantifies the overshoot during the aggressive recovery.

**Table 7:** Descriptive Statistics for Process Variables (Outflow Increase Disturbance).

	Level	Control Sig.	Setpoint	Error	Valve Clos.
**Count**	61193	61193	61193	61193	61193
**Mean (%)**	49.97	20.66	50.00	0.03	45.00
**Std (%)**	24.46	4.75	24.49	1.01	5.00
**Min (%)**	1.96	0.00	10.00	−30.29	40.00
**Max (%)**	90.98	100.00	90.00	15.39	50.00

**Fig. 13:**
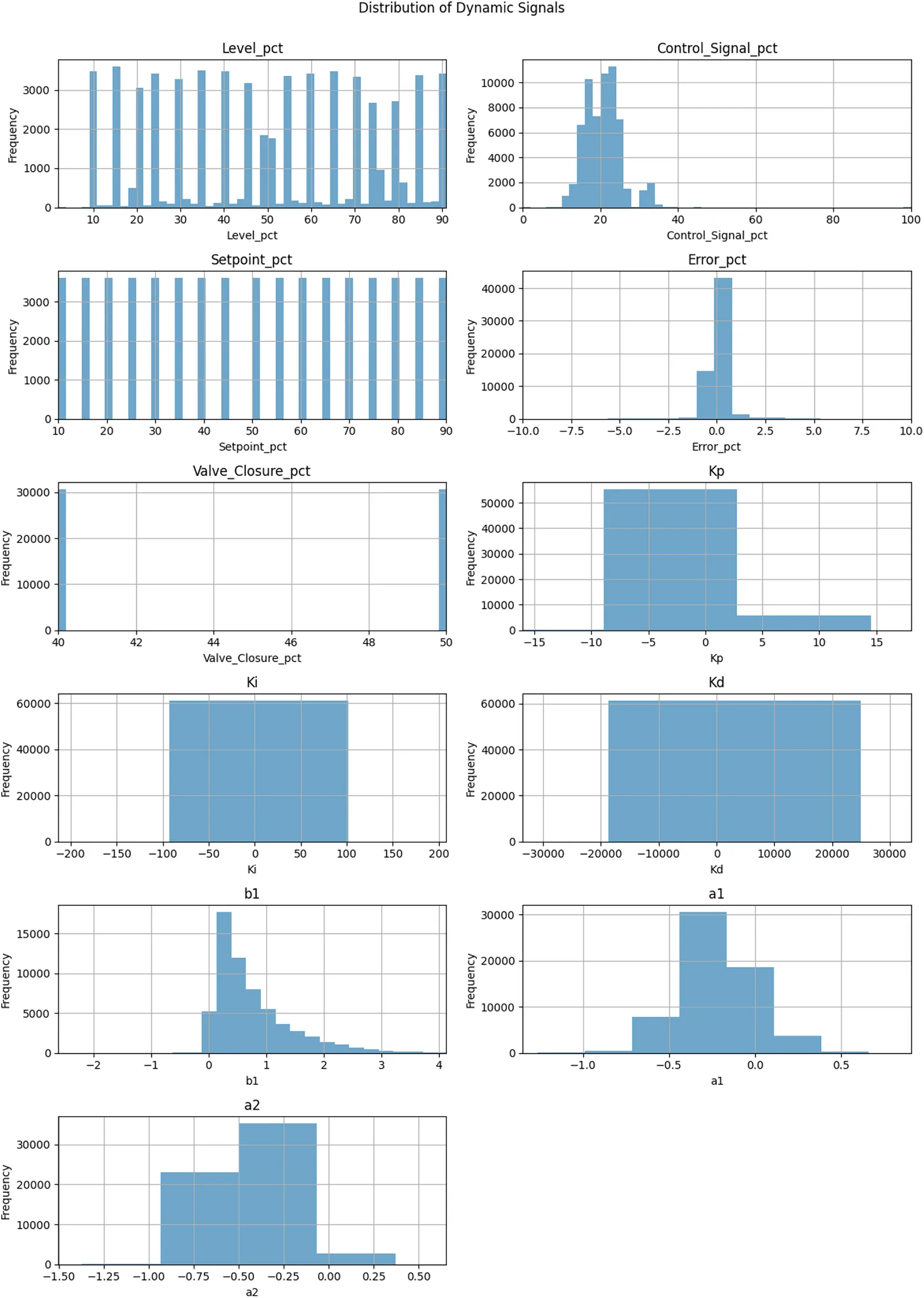
Distribution of dynamic signals collected during the Outflow Increase Disturbance experiment.

The corresponding distributions of all dynamic signals for this experiment are shown in Fig. 13.

#### Validation of Adaptive Response and Stability

The parameter statistics in Table 8 validate the RLS algorithm’s behavior. The standard deviation values are significantly lower than those observed in the saturated experiments (see Table 6), reflecting that the system operated in a stable linear regime for the majority of the time. However, the min/max values confirm that the algorithm reacted strongly during the transient event to adapt the model to the new hydraulic condition.

**Table 8:** Descriptive Statistics for Model Parameters (Outflow Increase Disturbance).

	Kp	Ki	Kd	b1	a1	a2
**Count**	61193	61193	61193	61193	61193	61193
**Mean**	1.01	−2.58	67.15	0.76	−0.22	−0.42
**Std**	3.40	42.19	6.70e3	0.67	0.23	0.22
**Min**	−349.6	−1259.5	−8.9e5	−7.54	−12.81	−8.78
**Max**	237.7	8460.7	1.29e6	5.26	0.93	13.00

### Analysis of Disturbance Rejection (Outflow Decrease)

These experiments validate the system’s response to a major state transition: moving from a challenging high-outflow condition (saturated) to a stable low-outflow condition. Similar to the previous section, zero-value readings caused by excessive turbulence were removed during post-processing to facilitate statistical analysis.

#### Validation of Multi-Stage Transient Response

**Fig. 14:**
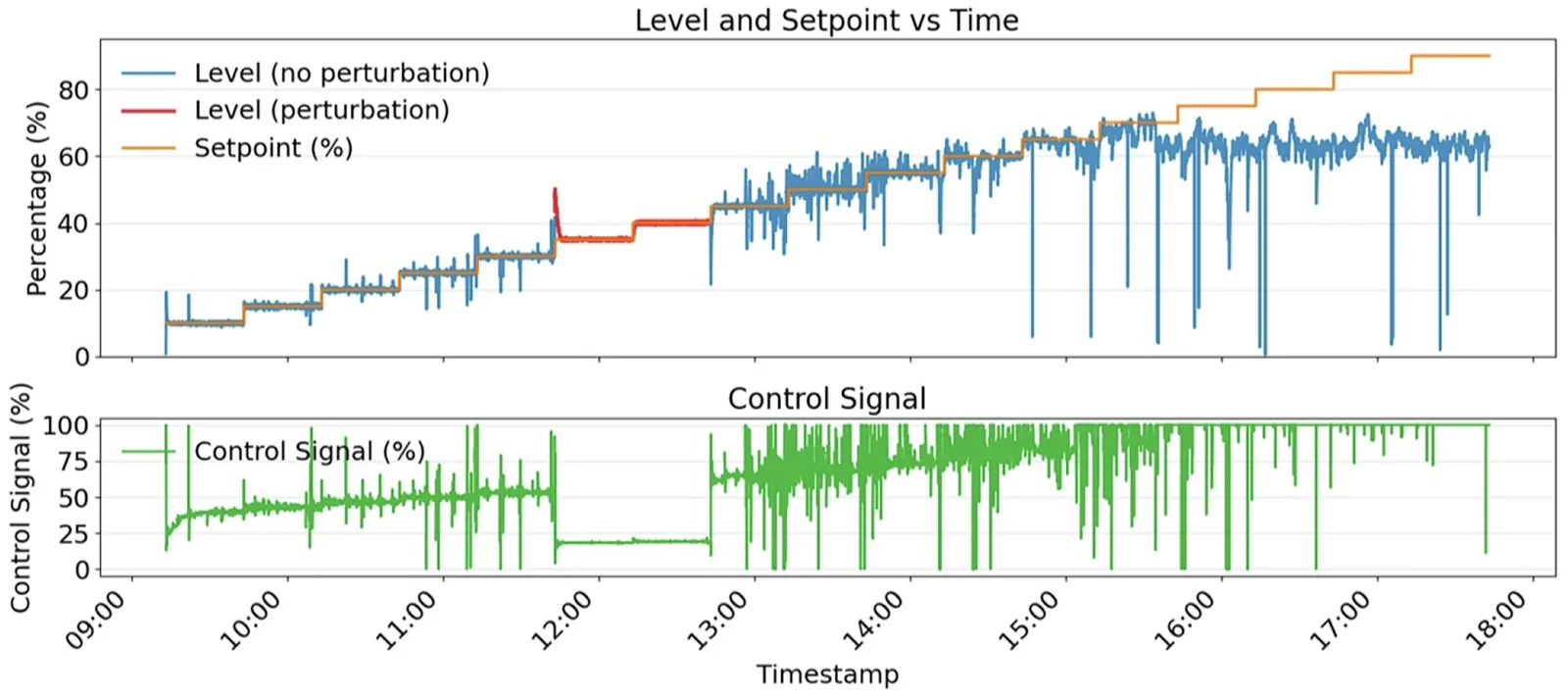
Time-series response during the Outflow Decrease Disturbance, showing the transition from saturation to stability.

Fig. 14 provides the primary visual evidence of this complex event, validating three distinct phases:

1. **Initial Saturation:** The system starts in a high-outflow state where the Level_pct fails to track the setpoint despite the control signal being saturated near 100%.
2. **The Disturbance:** The sudden reduction in outflow causes the level to rise sharply, creating a large negative error.
3. **Stabilization:** The controller reacts by dropping the signal to almost 0% before settling at a new, stable, non-saturated value.

Table 9 quantifies this behavior. Unlike previous experiments, the mean error (4.12%) and standard deviation (8.81%) are high, validating that the system spent a significant portion of the experiment in a non-nominal state.

**Table 9:** Descriptive Statistics for Process Variables (Outflow Decrease Disturbance).

	Level	Control Sig.	Setpoint	Error	Valve Clos.
**Count**	60523	60523	60523	60523	60523
**Mean (%)**	45.81	68.83	49.93	4.12	85.00
**Std (%)**	19.87	26.69	24.50	8.81	5.00
**Min (%)**	0.39	0.00	10.00	−19.02	80.00
**Max (%)**	81.96	100.00	90.00	88.04	90.00

#### Validation of Adaptive Response and Bimodal Behavior

**Fig. 15:**
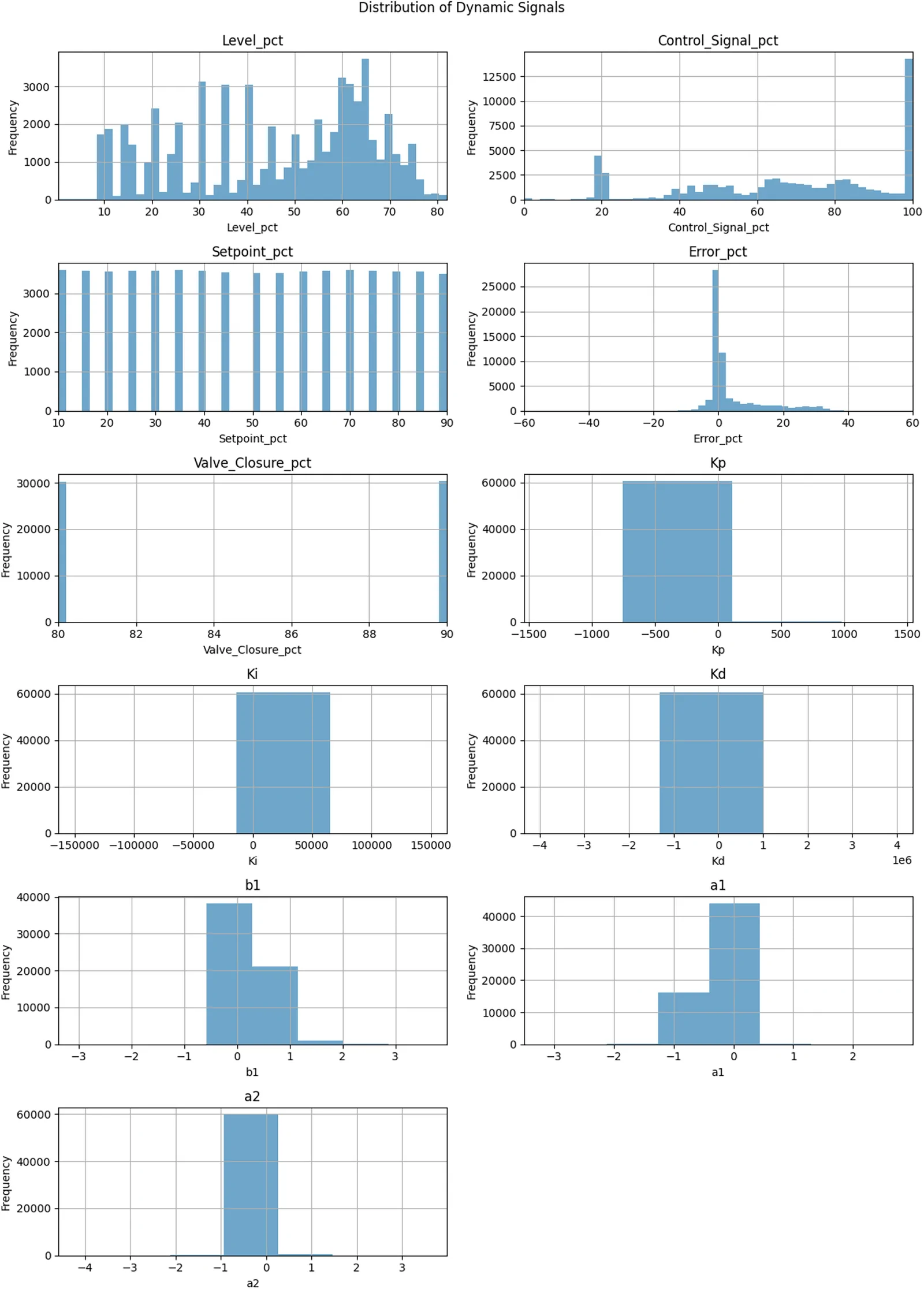
Distribution of dynamic signals collected during the Outflow Decrease Disturbance experiment, highlighting the bimodal control action.

The histograms in Fig. 15 confirm the dual nature of this experiment. The Control_Signal_pct distribution is visibly bimodal, validating that the dataset correctly captures two distinct operating regimes within a single time-series.

Regarding the model parameters (Table 10), the extreme max/min values reflect the difficulty of the initial saturated phase. However, the system’s ability to transition to a stable controller output validates the robustness of the adaptive mechanism even after prolonged saturation.

**Table 10:** Descriptive Statistics for Model Parameters (Outflow Decrease Disturbance).

	Kp	Ki	Kd	b1	a1	a2
**Count**	60523	60523	60523	60523	60523	60523
**Mean**	2.78	−95.51	2.06e3	0.30	−0.25	−0.30
**Std**	154.3	1.64e4	4.35e5	0.37	0.33	0.43
**Min**	−3.5e4	−3.6e6	−1.5e7	−19.62	−20.88	−30.77
**Max**	8.8e3	3.03e5	1.01e8	23.65	21.77	28.91

### Discussion of Dataset Validity

The analysis of the 25 datasets reveals consistent patterns in both the physical plant’s behavior and the adaptive controller’s response. These observations support the validity and internal consistency of the dataset. The key findings are summarized below.

#### Physical Regimes and Actuator Saturation

A crucial insight emerges from comparing the ‘Baseline’ and ‘Constant Passive Outflow’ experiments. The data validates the existence of two distinct operating regimes:

- **Laminar:** In the ‘Baseline’ configuration, the system operates in a regime where the controller achieves high-precision tracking.
- **Turbulent:** The high-outflow configuration forces the system into a saturated state.

This is most clearly evidenced by the ‘Outflow Decrease’ disturbance experiment. The bimodal distribution of the control signal validates that the plant transitions from a saturated, noisy state to a stable, controllable state within a single run. This confirms that the observed tracking difficulties are not algorithmic failures, but consequences of physical limitations: pump saturation (unable to overcome passive outflow) and turbulence-induced sensor noise (degrading the signal-to-noise ratio).

#### Adaptive Parameter Variability

The adaptive parameters (Kp, Ki, Kd) exhibited high variance and extreme outliers, particularly in the saturated experiments. Two factors contribute to this behavior:

- **Numerical Sensitivity:** The Dahlin synthesis equations involve divisions by identified parameters, so small variations near zero can produce large gain fluctuations.
- **RLS Behavior under Noise:** With a forgetting factor of *λ*_*RLS*_ = 0.9, the algorithm responds rapidly to measurement variations, including sensor noise.

This variability is documented in the dataset and may be of interest for researchers studying adaptive algorithm robustness.

#### Negative Gain Values

The negative mean values observed for Ki and Kd are a consequence of the discrete-time model structure and the Dahlin synthesis equations. This is mathematically consistent with the identified pole locations and does not indicate controller instability. Practical stability was maintained throughout all experiments due to the actuator saturation limits (0–100%), which constrained the physical control signal regardless of computed gain values.

### Baseline Anomaly Detection Analysis

Beyond benchmarking control strategies, the datasets are highly valuable for developing data-driven monitoring algorithms, specifically for Anomaly Detection. To validate this use case, a One-Class SVM (OC-SVM) was implemented to detect anomalies by learning the boundary of “normal” operation^12,13^.

Unlike standard supervised classification, the OC-SVM was trained exclusively using data from the 10 ‘Steady-State Baseline’ experiments. This setup mimics a realistic industrial scenario where fault data is often unavailable during the training phase. The trained model was then tested on a completely unseen dataset: the ‘Outflow Increase’ disturbance experiment conducted with a 50% controllable valve opening.

**Fig. 16:**
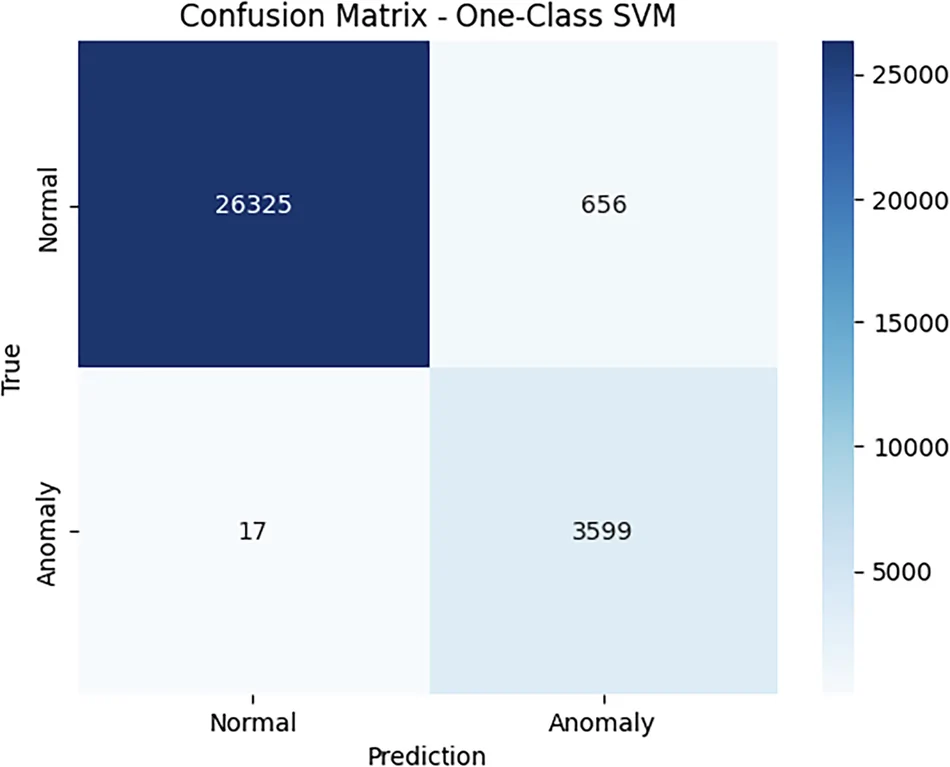
Confusion Matrix for the One-Class SVM model. The model was trained exclusively on nominal ‘Baseline’ data and tested on the ‘Outflow Increase’ (50% valve) experiment.

The results, summarized in Table 11 and Figure 16, demonstrate the dataset’s high quality for anomaly detection tasks. The model achieved an overall accuracy of 98%, with a Recall of 1.00 for the ‘Anomaly’ class, correctly identifying 3599 out of 3616 anomalous samples. While the Precision for anomalies is slightly lower (0.85) due to some false positives, this trade-off is typical in safety-prioritized one-class classification. This analysis confirms that the ‘Baseline’ data possesses a consistent, learnable signature, and the ‘Disturbance’ experiments provide distinct, detectable deviations, validating the collection as a robust testbed for monitoring research.

**Table 11:** Classification Report for One-Class SVM (Anomaly Detection).

Class	Precision	Recall	F1-Score	Support
Normal	1.00	0.98	0.99	26981
Anomaly	0.85	1.00	0.91	3616
**Accuracy**			**0.98**	**30597**
**Macro Avg**	0.92	0.99	0.95	30597
**Weighted Avg**	0.98	0.98	0.98	30597

## Usage Notes and Limitations

This dataset is designed to support research in adaptive control, disturbance rejection, and data-driven monitoring. Users should consider the following points when working with the data:

### Signal Normalization

All percentage-scaled variables (Level_pct, Control_Signal_pct, Setpoint_pct, Valve_Closure_pct, Error_pct) are expressed as percentages of their respective full-scale ranges: 0–25 cm for level, 0–10 V (mapped to 0–50 Hz pump frequency) for control signal, and 0–100% valve aperture. This normalization facilitates direct comparison across experiments but requires rescaling for applications requiring physical units.

### Excitation Limitations

The experimental design employs staircase setpoint profiles under closed-loop control. While this approach effectively explores different operating points and captures realistic controller–plant interactions, it does not guarantee persistence of excitation. Consequently, the dataset may have limited informativeness for offline system identification methods that require rich frequency content or for identifying high-frequency dynamics. Users interested in system identification should consider these data as representative of closed-loop operating conditions rather than optimally excited identification experiments.

### Measurement Uncertainty

Under turbulent hydraulic conditions (particularly in the Passive Outflow experiments), the ultrasonic sensor measurements exhibit increased noise due to water surface oscillations. Zero-level readings in some turbulent experiments represent sensor artifacts rather than actual empty-tank conditions.

### Recommended Applications

The dataset is particularly well-suited for: (1) benchmarking adaptive control algorithms under varying operating conditions, (2) evaluating disturbance rejection strategies, (3) developing and testing anomaly detection methods, (4) studying actuator saturation effects, and (5) control-oriented identification using closed-loop data.

## Data Availability

The complete dataset generated and analyzed during the current study is publicly available in the Zenodo repository^11^ under the Digital Object Identifier https://doi.org/10.5281/zenodo.17688566. The data are provided under the Creative Commons Attribution 4.0 International (CC BY 4.0) license, allowing for unrestricted use, distribution, and reproduction in any medium, provided the original work is properly cited.

## Code availability

The complete source code developed for this study is hosted on GitHub. This includes:

• The MATLAB/Simulink models and Arduino firmware used for real-time data acquisition and adaptive control.

• The Python scripts utilized for the Exploratory Data Analysis (EDA) and the One-Class SVM anomaly detection validation.

The repository can be accessed at: https://github.com/pedropgg/TwoTank-Dataset-Code.
